# Decomposition of socioeconomic inequalities in glaucoma knowledge in Taiwan

**DOI:** 10.4178/epih.e2024004

**Published:** 2023-12-05

**Authors:** Chiun-Ho Hou, Christy Pu

**Affiliations:** 1 Institute of Public Health, National Yang Ming Chiao Tung University School of Medicine, Taipei, Taiwan; 2 Department of Ophthalmology, National Taiwan University Hospital, Taipei, Taiwan

**Keywords:** Socioeconomic disparities, Glaucoma knowledge, Concentration index, Decomposition, Inequality

## Abstract

**OBJECTIVES:**

Glaucoma knowledge is strongly associated with medication adherence and preventive behavior. Studies have frequently reported socioeconomic inequalities in glaucoma knowledge. This study aimed to decompose such inequalities. Decomposition analysis enables the design of policies directly targeting the underlying causes of inequality.

**METHODS:**

We performed a cross-sectional survey from January 1, 2019 to June 30, 2019, at the departments of ophthalmology of 2 medical centers belonging to a hospital chain in northern Taiwan. Socioeconomic inequalities in glaucoma knowledge were ranked based on 3 aspects of socioeconomic status (SES): (1) education, (2) income, and (3) self-perceived financial status. The concentration index was calculated and decomposed using decomposition analysis. Elasticity and marginal effects were estimated for each decomposed factor.

**RESULTS:**

In total, 1,203 patients completed the survey. Both measures of glaucoma knowledge and overall glaucoma knowledge score significantly contributed to the progressivity of knowledge inequalities (pro-high-SES inequalities). The concentration index for overall knowledge score with respect to education was 0.166 (p<0.001). Both objective and subjective measures of SES were associated with pro-high-SES inequalities. Our decomposition analysis revealed that demographic factors and attitudinal factors such as the level of concern regarding developing glaucoma contributed significantly to SES-based inequalities in glaucoma knowledge.

**CONCLUSIONS:**

Our decomposition analysis provided empirical evidence regarding the underlying causes of SES-based inequalities in glaucoma knowledge. Efforts to improve glaucoma knowledge should consider specific factors that drive SES-based inequalities, such as age, sex, and concern about vision health, to ultimately achieve low SES-based inequalities.

## INTRODUCTION

Inequalities in vision health have often been attributed to socioeconomic disparities. The global burden of glaucoma has been increasing over the years, and people of lower socioeconomic standing experience a greater glaucoma burden [1]. Low income and lack of health insurance coverage are associated with unmet needs for cataract surgery [2]. In the United States, people in poverty or with lower income have been reported to be significantly less likely to make ophthalmology visits [3,4]. The prevalence of blindness has also been shown to be higher among people with lower income [5].

Glaucoma is the second leading cause of blindness worldwide, and visual field status deterioration is strongly associated with vision-related quality of life [6]. Although glaucoma has severe consequences, people at risk of glaucoma are often unaware of the risks or symptoms of the disease [7-9]. Knowledge of glaucoma is thus essential to treatment adherence [10-12]. Health literacy has also been shown to be strongly associated with adherence to glaucoma therapy [13]. Accordingly, education about the symptoms of glaucoma and visual impairment can be a vital first step in promoting proactive ophthalmic health care [14]. However, studies have often reported unsatisfactory levels of glaucoma knowledge, particularly among patients with glaucoma [15,16] and the public [17].

Policymakers and researchers’ interest in glaucoma knowledge has evolved beyond a simple understanding of the positive relationship between glaucoma knowledge and patient outcomes; such interest has shifted toward identifying the underlying causes of knowledge inequality. Nevertheless, addressing socioeconomic problems that affect glaucoma knowledge can be difficult. To overcome this difficulty, interventions specifically targeting underlying factors are likely to be helpful.

Studies have addressed the relationship between lower socioeconomic status (SES) and poor glaucoma knowledge. Hoevenaars et al. [16] demonstrated that poor glaucoma knowledge was associated with low education levels. A study conducted in Ghana revealed that higher education levels were associated with greater awareness of glaucoma, although awareness is not equivalent to accurate glaucoma knowledge [18]. A study performed in Mexico determined that individuals with higher education levels were approximately 4 times more likely to have accurate glaucoma knowledge [19]. In general, health literacy is lower among individuals with lower SES, and low health literacy may explain SES-based health disparities [20]. Low SES has been reported to be associated with a greater prevalence of glaucoma as well as a greater severity of glaucoma at presentation [21,22], and this is attributable to lower glaucoma knowledge in individuals of lower SES.

Studies have yet to decompose socioeconomic inequalities in glaucoma knowledge in order to identify their underlying factors. Decomposing such inequalities is essential for policy-making because it can provide empirical evidence on factors that can mitigate or exacerbate the inequalities.

The aim of the present study was to measure socioeconomic inequalities in glaucoma knowledge by using the concentration index. The concentration index is often utilized to measure disparities in variables associated with SES [23]. We then decomposed such inequalities to identify their underlying factors. Our study is the first to decompose socioeconomic inequalities in glaucoma knowledge. Accordingly, this study contributes to the literature on glaucoma knowledge inequalities by providing evidence on the distribution of glaucoma knowledge across socioeconomic gradients. This study also expands the literature on glaucoma knowledge inequalities by establishing the contribution of socioeconomic inequalities to glaucoma knowledge inequalities.

## MATERIALS AND METHODS

### Data

#### Survey data

We performed a cross-sectional survey from January 1, 2019 to June 30, 2019, at the ophthalmology departments of 2 medical centers belonging to a hospital chain in northern Taiwan. Patients who sought care from 4 designated physicians at the ophthalmology departments during the study period were approached and invited to participate in the study. For this survey, we designed a questionnaire to collect information on the participants’ characteristics and their glaucoma knowledge. Specifically, the questionnaire included sections on the respondents’ demographic characteristics and SES and their knowledge of the causes and symptoms of glaucoma (as explained in the Measures section). The questionnaire was reviewed by 6 ophthalmologists and 2 public health experts. The questionnaire was pretested. To assess questionnaire reliability, 15 patients from the pretest were asked to complete the questionnaire again upon their next visit.

In the final survey, all participants were provided copies of the questionnaire in paper format, and they completed the questionnaires by themselves. Trained assistants were present to answer any questions the respondents might have while completing the questionnaires.

#### National Health Insurance claims data

Taiwan implemented a National Health Insurance (NHI) program in 1995. This program provides coverage for essential medical care and pharmaceuticals, thus eliminating most financial barriers to medical care. We linked our survey data to NHI claims data to obtain information about the number of ophthalmology visits within 1 year before the survey as well as information about insurable income. The inclusion of the number of ophthalmology visits served as a proxy for recent visits to ophthalmologists, which was identified as a predictor for awareness of glaucoma as a leading cause of blindness in the study conducted by Ichhpujani [14]. It is important to note, however, that Ichhpujani’s study focused on 119 staff members from a tertiary hospital, who may have had significantly different characteristics from the participants in our own study.

### Socioeconomic status measures

#### Education (objective SES measure)

Education level was self-reported by the respondents and was considered a categorical variable with the following categories: (1) primary school or illiterate, (2) junior high school, (3) senior high school, and (4) college and above. The original categorical response was converted to corresponding years of education and was analyzed as a continuous variable.

#### Income (objective SES measure)

Because income is often misreported in surveys and because non-response is not uncommon [24], we recorded income as the insurable income from the NHI database. This income measure was thus free from reporting bias and non-response bias. Income was treated as a continuous variable.

#### Satisfaction with current financial status (subjective SES measure)

We also employed subjective financial status measures. Several studies have documented the importance of using subjective SES in health disparity studies [25,26]. Accordingly, in our survey, we measured subjective SES by using the following question: “How would you rate your household financial status?” Answers to the question were as follows: “lots of surplus,” “some surplus,” “balanced,” “some shortages,” and “considerable shortage.”

### Glaucoma knowledge

We used 2 measures of glaucoma knowledge—namely, general knowledge on glaucoma and knowledge on glaucoma risk factors. We designed items to measure such knowledge based on the relevant literature. Each item had 3 response options: (1) “true,” (2) “false,” and (3) “don’t know.” The third option (“don’t know”) was included to prevent participants from accidentally guessing the correct response. General knowledge on glaucoma was measured using the following items: (1) “Glaucoma can be completely cured,” (2) “Cataract can lead to glaucoma,” (3) “Overuse of the eyes can lead to glaucoma,” (4) “People with no family history have lower chances of getting glaucoma,” (5) “Early-stage glaucoma does not have symptoms,” and (6) “Generally speaking, ocular pressure lower than 30 mmHg is considered normal.” Knowledge on glaucoma risk factors was measured according to the respondents’ responses to whether any of the following items are risk factors for glaucoma: (1) genetics, (2) high intraocular pressure, (3) high myopia or hyperopia, (4) ocular trauma, (5) long-term use of steroids, (6) diabetes, (7) hypertension, (8) obesity, (9) lack of exercise, (10) unbalanced diet, (11) overuse of electronic devices, and (12) lack of sleep.

### Concentration index

The concentration index is commonly employed to gauge inequalities in a specific variable that is linked to SES [23]. The concentration index Cx can be derived as follows [27]:


$$Cx\left(W|S\right)=\frac{2cov\left(E_{i},P_{i}\right)}{\bar{E}}=\frac{1}{n}\sum_{i=1}^{n}\left\{\frac{E_{i}}{\bar{E}}\left(2P_{i}-1\right)\right\}$$

Where *E_i_* is the variable for which an inequality is measured, *S* is the socioeconomic variable to be investigated, and *P_i_* is the fractional socioeconomic rank. *C* ranges from (1−*n*)/*n*—representing maximal pro-low-SES inequalities—to (*n*−1)/*n*, representing maximal pro-high-SES inequalities [27].

We used the method proposed by Kakwani [28] to decompose inequalities in glaucoma knowledge scores according to individual SES. In this method, factors that might be associated with an inequality (denoted *x*) are analyzed. Total glaucoma knowledge scores are expressed as a linear function of *x*. The *Cx* value is decomposed into the sum of *px*, where *px* represents the product of the elasticity of glaucoma knowledge with respect to *x* and the degree to which such knowledge is unevenly distributed across the SES variable of interest (as measured by the concentration index for x) [29]. Elasticity represents the sensitivity of SES-based inequalities in glaucoma knowledge to changes in the concentration index of a given factor. Subsequently, we defined a residual component for inequalities that could not be explained by the regressors. To implement the decomposition analysis, we used linear regression to estimate the association between the glaucoma knowledge score and the explanatory variables. The covariates included age, sex, marital status (never married, married/cohabiting, widowed, separated), self-rated financial status, place of residence (Taipei, others), employment status (employed, unemployed, retired), self-rated health, weight (underweight, normal weight, overweight, obese), smoking status (never smoker, smoker, ex-smoker), glaucoma (yes/no), cataract (yes/no), self-perceived glaucoma risk (very high risk, average risk, low risk, no idea, already have glaucoma/blind), concern about current vision health (very worried, worried, not worried), and number of outpatient ophthalmology visits in the past year. We selected our final model in accordance with Akaike’s information criterion [30].

### Ethics statement

The study was approved by the Institutional Review Boards (IRBs) of National Yang-Ming University and the two medical centers involved in the study (IRB approval No. YM109014E). Informed consent was confirmed by the IRB.

## RESULTS

Table 1 presents participants’ characteristics stratified by their mean education level. Participants with <10 years of education were older and less likely to be males than were those with > 10 years of education. Participants with < 10 years of education were also less likely to report having surplus household income and had significantly lower income than those with > 10 years of education. Thus, the 3 SES variables were highly correlated.

Participants with lower education levels were significantly less likely to be employed than those with higher education levels, possibly because of their older age. Participants with lower education levels were more likely to report themselves as having poor health. However, they were more likely to have never smoked. They were also more likely to report that they had no idea about their risk of developing glaucoma and report that they were not worried about vision health.

**Figure 1.**
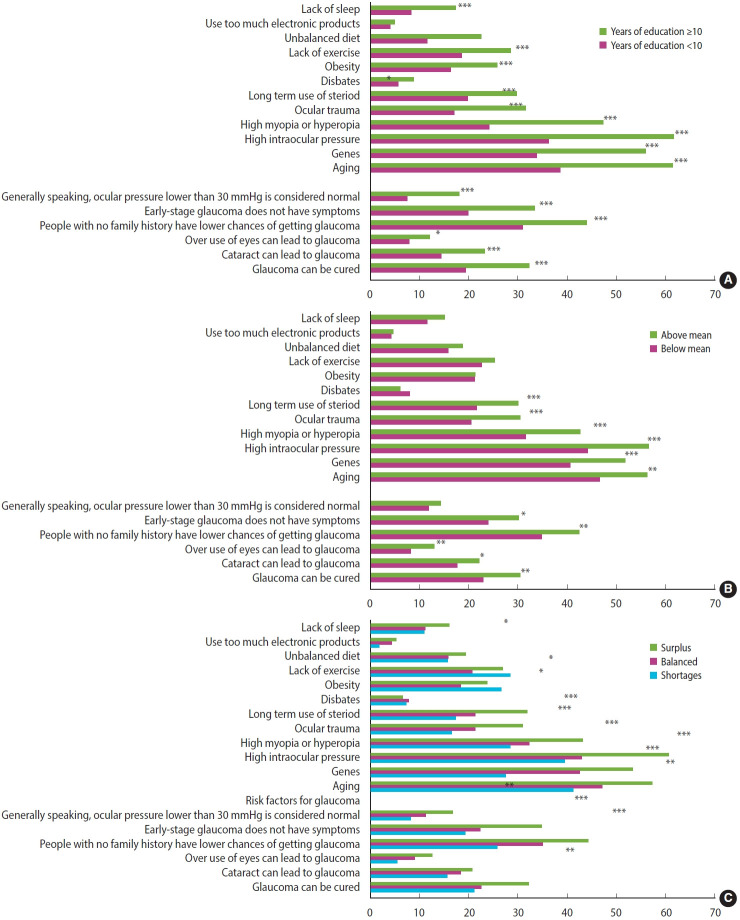
Glaucoma knowledge by (A) education, (B) income, and (C) self-rated financial status. *p<0.05, **p<0.01, ***p<0.001.

Regarding glaucoma risk factors, we observed that the proportion of correct responses to the questions differed significantly by education level (Figure 1A). The proportion of correct responses to all questions was higher among participants with higher education level. However, the proportion of participants with correct answers was generally low, regardless of years of education. For participants with > 10 years of education, the question as to whether high intraocular pressure is a risk factor for glaucoma had the highest proportion of correct responses. However, only 61.6% of the participants with > 10 years of education answered this question correctly, and only 36.2% of those with fewer years of education answered this question correctly.

Regarding general knowledge of glaucoma, less than 20% of the participants knew what the normal intraocular pressure range is, regardless of education level. This may be relatively unsurprising because this was considered one of the more difficult questions; nevertheless, the participants also answered poorly on easier questions such as whether early stages of glaucoma have symptoms.

The distributions of glaucoma knowledge by income and self-reported financial status (Figure 1B and C) exhibited similar patterns. For self-reported financial status, we combined the “lots of surplus” and “some surplus” categories and combined the “considerable shortage” and “some shortage” categories to ensure a reasonable number of participants in each category. We noted a clear dose-response relationship between glaucoma knowledge score and self-reported financial status; participants with higher self-reported financial status were more likely to provide correct responses.

Table 2 presents the concentration index values derived for the glaucoma knowledge measures according to the various SES measures. A positive concentration index value was considered to indicate progressive (pro-high-SES) inequality, whereas a negative value was considered to indicate regressive (pro-low-SES) inequality. Education level was associated with the largest progressivity of inequalities in glaucoma knowledge, indicating a significant inequality favoring participants with higher education levels. We also observed progressivity in all knowledge scores when the participants were ranked by income and self-reported financial status, although the corresponding magnitudes were smaller than those observed when the participants were ranked by years of education.

Table 3 presents the results of the decomposition of SES-based inequalities for overall glaucoma knowledge score. We observed that age was a major contributor to inequalities based on education, accounting for approximately 17% of the inequalities observed. Age was thus determined to be a factor exacerbating pro-high-SES-based inequalities in glaucoma knowledge. We obtained similar results for inequalities based on income. However, age contributed only 2.2% of inequalities based on self-perceived financial status.

Sex contributed negatively to inequalities based on all 3 SES measures. This means that sex mitigated pro-high-SES inequalities. However, sex contributed approximately 9% of inequalities based on education level and only 1.7% of inequalities based on self-reported financial status. Marital status also contributed negatively to progressivity; it accounted for approximately 18% of inequalities based on education level.

Self-reported financial status and income accounted for 5.6% of the progressivity observed for inequalities based on education level. This signifies that self-rated financial status and income are not major contributors to education-based glaucoma knowledge inequalities. However, education level accounted for 75.4% and 58.6% of the progressivity observed for inequalities based on self-reported financial status and income, respectively.

Self-perceived glaucoma risk and concern about vision health together accounted for 5.6% of inequalities based on education level and accounted for 4.3% of inequalities based on income, but they accounted for only 0.3% of inequalities based on subjective SES. The number of outpatient ophthalmology visits in the past year was not a major contributor to glaucoma knowledge inequalities based on all 3 SES measures.

## DISCUSSION

In this study, we analyzed socioeconomic inequalities in glaucoma knowledge and decomposed them using different SES measures. Our major findings are outlined as follows: (1) Both measures of glaucoma knowledge and overall knowledge score exhibited significant progressivity (i.e., pro-high-SES inequalities). (2) Pro-high-SES inequalities were observed for both objective and subjective SES measures. (3) Although the 3 SES measures were positively correlated, factors accounting for SES-based glaucoma knowledge inequalities differed significantly for the various SES measures.

This study has several strengths. First, previous studies have not decomposed the factors explaining SES-based glaucoma knowledge inequality; thus, designing policies to reduce this SES-based disparity is extremely difficult. Only by knowing the factors behind this disparity can policies be designed to address the factors directly. We tested a wide range of SES measures, making our results useful for policy design for subpopulations where one SES measure may be more important for measuring inequality than others. Second, we tested 2 types of glaucoma knowledge, making it possible to provide a detailed breakdown of the types of glaucoma knowledge subject to SES-based inequality.

We identified that among the 3 SES measures, education level consistently accounted for the largest proportion of SES-based inequalities in glaucoma knowledge. Education level still accounted for a considerable proportion of such inequalities based on income and subjective SES. This signifies that regardless of the SES measures used, improving glaucoma knowledge in individuals with relatively low education levels could be an effective disparity-reducing strategy. Individuals with higher education levels may have greater cognitive abilities, 31 allowing them to gain glaucoma knowledge more easily. Such cognitive abilities may not be associated with higher self-rated financial status or higher income. Accordingly, future studies on SES-based disparities in health literacy should not ignore the importance of disparities in education.

We found that age contributed positively to the progressivity of SES-based inequalities in glaucoma knowledge. A possible explanation for this finding is that older people have more experience with ophthalmic conditions. This thus validates the necessity of increasing education programs on ophthalmic conditions for younger patients. A United States study reported that poorer glaucoma medication adherence was more common in patients aged < 50 years or ≥ 80 years [11]. Another study also revealed that very young and very old patients had relatively low glaucoma knowledge [12]. Accordingly, providing glaucoma education to these individuals can help reduce socioeconomic inequalities in glaucoma knowledge.

The number of outpatient ophthalmology visits in the past year did not have a considerable contribution to glaucoma knowledge inequalities based on all 3 SES measures. Previous studies have shown that visits to an eye practitioner or receiving eye examination were associated with higher glaucoma knowledge or awareness [14,32]. Similarly, our results suggest that under a universal health coverage system such as Taiwan’s NHI, visits to an ophthalmologist are unlikely to be correlated with income or other SES measures and would thus not contribute to SES-based inequalities in glaucoma knowledge. SES is likely to affect inequalities in glaucoma knowledge through other factors.

We also analyzed whether concern about vision health and self-perceived risk of glaucoma explained SES-based inequalities in glaucoma knowledge. Although these factors accounted for only a moderate proportion of the observed SES-based inequalities, they exacerbated such inequalities. A United States study reported that participants who frequently failed to attend appointments at a glaucoma clinic were more likely to perceive their condition as less severe than those who attended scheduled appointments [33]. Accordingly, on the basis of our findings and those of previous studies, we suggest that to reduce SES-based glaucoma knowledge inequalities, authorities should provide information clarifying the risk of poor vision health among individuals of low SES. For example, individuals of low SES should be provided with information on the likelihood of developing glaucoma. Moreover, individuals with a high risk of glaucoma can be educated on the danger of ignoring this risk.

This study has several limitations. First, the participants were selected from a single hospital chain; hence, the results may not be generalizable to the entire country. However, this hospital chain is the largest in Taiwan; therefore, our sample has some degree of representativeness. Second, the sample size was limited by funding availability, which may have affected the study’s statistical power. Third, although we used administrative data to measure income, income may have been mismeasured. For example, participants may have had income that was not recorded in the administrative system. Nevertheless, we believe the income measure we used should capture income more accurately than self-report measures.

Glaucoma knowledge is vital to glaucoma prevention and treatment adherence. Implementing policies aimed at reducing SES-based inequalities in glaucoma knowledge is crucial for the achievement of equity in vision health. We decomposed inequalities in glaucoma knowledge based on SES in order to identify factors contributing to such inequalities. We observed that education level had a major contribution to SES-based inequalities in glaucoma knowledge. Our findings provide valuable information that could be useful for improving outcomes for patients with glaucoma. Additionally, efforts toward improving glaucoma knowledge should consider specific factors that contribute to poor glaucoma knowledge in individuals of lower SES.

## Figures and Tables

**Table 1.** Participant characteristics (n=1,203)

Characteristics	Years of education		p-value
	<10 yr (n=583)	≥10 yr (n=620)	
Age (yr)	72.6±8.33	53.1±14.67	<0.001
Sex (male)	135 (23.2)	331 (53.4)	<0.001
Marital status			<0.001
Never married	22 (3.8)	168 (27.1)	
Married/cohabiting	441 (75.6)	450 (72.6)	
Widowed/separated	120 (20.6)	2 (0.3)	
Self-rated financial status			<0.001
Lots of surplus	1 (0.2)	32 (5.2)	
Some surplus	108 (18.5)	313 (50.5)	
Balanced	400 (68.6)	240 (38.7)	
Some shortage	63 (10.8)	32 (5.2)	
Considerable shortage	11 (1.9)	3 (0.5)	
Place of residence (Taipei=1)	167 (28.6)	175 (28.2)	0.872
Employment			<0.001
Employed	25 (4.3)	396 (63.9)	
Unemployed	24 (4.1)	35 (3.6)	
Retired	534 (91.6)	189 (30.5)	
Self-rated health			<0.001
Very good	9 (1.5)	22 (3.5)	
Good	54 (9.3)	135 (21.8)	
Average	138 (23.7)	227 (36.6)	
Poor	282 (48.4)	203 (32.7)	
Very poor	100 (17.1)	33 (5.3)	
Weight			0.140
Underweight	20 (3.4)	33 (5.3)	
Normal weight	354 (60.7)	388 (62.6)	
Overweight	171 (29.3)	172 (27.7)	
Obese	38 (6.5)	27 (4.3)	
Smoking status			<0.001
Never smoked	502 (86.1)	445 (71.8)	
Smoke	21 (3.6)	57 (9.2)	
Ex-smoker	60 (10.3)	118 (19.0)	
Glaucoma (yes=1)	89 (15.3)	103 (16.6)	0.524
Cataract (yes=1)	350 (60.0)	214 (34.5)	<0.001
Self-perceived glaucoma risk			0.001
Very high risk	10 (1.7)	12 (1.9)	
Average risk	7 (1.2)	22 (3.5)	
Low risk	155 (26.6)	204 (32.9)	
No idea	346 (59.3)	297 (42.9)	
Already have glaucoma/blind	65 (11.1)	85 (13.7)	
Worry about current vision health			<0.001
Very worried	72 (12.3)	80 (12.9)	
Worried	215 (36.9)	305 (49.2)	
Not worried	296 (50.8)	235 (37.9)	
Insurable income	27,066.64±27,987.98	40,371.45±35,078.34	<0.001
Annual outpatient ophthalmology visits	3.70±4.27	2.94±4.46	0.002

Values are presented as number (%) or mean±standard deviation.

**Table 2.** Concentration index based on different socioeconomic status measures

Variables	Concentration index	Standard error	p-value
Years of education			
General glaucoma knowledge	0.159	0.017	<0.001
Knowledge of glaucoma risk factors	0.168	0.014	<0.001
Overall glaucoma knowledge score	0.166	0.014	<0.001
Income (1,249-182,000 New Taiwan dollars)			
General glaucoma knowledge	0.087	0.017	<0.001
Knowledge of glaucoma risk factors	0.070	0.015	<0.001
Overall glaucoma knowledge score	0.075	0.014	<0.001
Satisfaction with current financial status			
General glaucoma knowledge	0.093	0.016	<0.001
Knowledge of glaucoma risk factors	0.075	0.013	<0.001
Overall glaucoma knowledge score	0.080	0.013	<0.001

**Table 3.** Decomposition of inequality in glaucoma knowledge by socioeconomic measures

SES measures	Marginal effect	Education		Self-perceived financial status		Income	
		Concentration index	% of contribution	Concentration index	% of contribution	Concentration index	% of contribution
Years of education	0.444	0.153	94.56	0.058	75.40	0.043	58.55
Self-rated financial status							
Surplus (reference)							
Balanced	-0.325	-0.161	3.72	-0.286	13.95	-0.018	0.92
Shortages	-0.496	-0.314	1.90	-0.909	11.58	-0.172	2.29
Income (mean, SD)	0.000	0.146	6.21	0.075	6.71	0.485	45.48
Age (mean, SD)	-0.018	-0.113	16.96	-0.007	2.22	-0.035	11.53
Sex (male)	0.652	-0.064	-8.95	-0.006	-1.73	-0.011	-3.50
Marital status							
Never married (reference)							
Married/cohabiting	0.487	-0.013	-0.62	0.025	2.56	0.029	3.08
Widowed/separated	1.583	-0.802	-17.15	-0.130	-5.83	-0.208	-9.78
Place of residence (Taipei=1)	0.190	-0.007	-0.07	0.041	0.78	0.044	0.88
Employment							
Employed							
Unemployed	0.124	0.079	0.06	-0.208	-0.36	-0.153	-0.28
Retired	0.201	-0.323	-5.21	-0.039	-1.31	-0.141	-5.01
Self-rated health							
Very good (reference)							
Good	-0.183	0.276	-1.05	0.171	-1.37	0.109	-0.91
Average	0.069	0.116	0.32	0.056	0.33	0.022	0.13
Poor	-0.082	-0.100	0.45	-0.064	0.61	-0.054	0.54
Very poor	0.340	-0.413	-2.07	-0.198	-2.08	-0.067	-0.74
Weight							
Underweight	-1.111	0.150	-0.97	0.080	-1.09	-0.009	0.13
Normal weight (reference)							
Overweight	-0.134	-0.043	0.22	-0.040	0.44	0.001	-0.01
Obese	-0.277	-0.155	0.31	-0.083	0.35	-0.007	0.03
Smoking status							
Never smoked (reference)							
Smoke	-0.466	0.226	-0.91	-0.126	1.07	-0.068	0.61
Ex-smoker	0.040	0.160	0.13	0.036	0.06	0.003	0.00
Glaucoma (yes=1)	1.128	-0.010	-0.25	-0.010	-0.48	-0.010	-0.51
Cataract (yes=1)	-0.181	-0.190	2.18	-0.016	0.39	-0.050	1.26
Self-perceived glaucoma risk							
High/medium	1.294	0.237	1.82	-0.011	-0.17	0.091	1.54
Worry about current vision health							
Very worried (reference)							
Worried	0.387	0.096	2.18	0.017	0.83	0.034	1.68
Not worried	-0.300	-0.093	1.63	0.011	-0.40	-0.027	1.04
Annual ophthalmology outpatient visits	-0.016	-0.088	0.61	-0.021	0.31	-0.062	0.95

SES, socioeconomic status; SE, standard error.
